# SimSpliceEvol2: alternative splicing-aware simulation of biological sequence evolution and transcript phylogenies

**DOI:** 10.1186/s12859-024-05853-z

**Published:** 2024-07-11

**Authors:** Wend Yam D. D. Ouedraogo, Aida Ouangraoua

**Affiliations:** Department of Computer Science, Université de Sherbrooke, 2500 Bd de l’université, Sherbrooke, QC J1K2R1 Canada

**Keywords:** Simulation, Exon-intron structure, Alternative splicing, Evolution, Transcript phylogeny

## Abstract

**Background:**

SimSpliceEvol is a tool for simulating the evolution of eukaryotic gene sequences that integrates exon-intron structure evolution as well as the evolution of the sets of transcripts produced from genes. It takes a guide gene tree as input and generates a gene sequence with its transcripts for each node of the tree, from the root to the leaves. However, the sets of transcripts simulated at different nodes of the guide gene tree lack evolutionary connections. Consequently, SimSpliceEvol is not suitable for evaluating methods for transcript phylogeny inference or gene phylogeny inference that rely on transcript conservation.

**Results:**

Here, we introduce SimSpliceEvol2, which, compared to the first version, incorporates an explicit model of transcript evolution for simulating alternative transcripts along the branches of a guide gene tree, as well as the transcript phylogenies inferred. We offer a comprehensive software with a graphical user interface and an updated version of the web server, ensuring easy and user-friendly access to the tool.

**Conclusion:**

SimSpliceEvol2 generates synthetic datasets that are useful for evaluating methods and tools for spliced RNA sequence analysis, such as spliced alignment methods, methods for identifying conserved transcripts, and transcript phylogeny reconstruction methods. The web server is accessible at https://simspliceevol.cobius.usherbrooke.ca, where you can also download the standalone software. Comprehensive documentation for the software is available at the same address. For developers interested in the source code, which requires the installation of all prerequisites to run, it is provided at https://github.com/UdeS-CoBIUS/SimSpliceEvol.

## Background

**Fig. 1:**
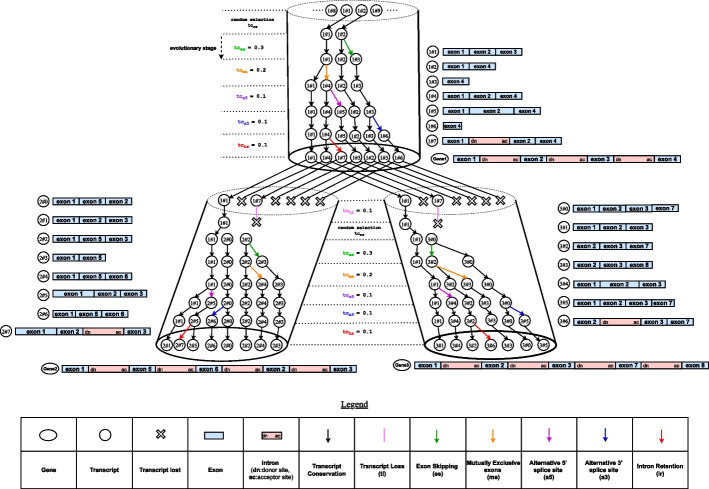
Illustration of the transcript evolution simulation framework. The figure depicts the phylogeny resulting from the simulated evolution of transcripts in a guide gene tree. The guide gene tree depicted as 3 cylinders consists in the evolution of two extant genes, Gene2 and Gene3, from an ancestral gene, Gene1. The bottom surfaces of the cylinders represent the two leaves (Gene2 and Gene3) of the guide gene tree and their ancestor (Gene1). The legend at the bottom of the figure shows the meaning for each graphical element. The exon-intron structures of each gene is diplayed, as well as the exon composition of each transcript. The evolution history consists of evolutionary stages. The root nodes of the transcript phylogeny correspond to transcript gains. The values of user input parameters are ($\texttt {tc\_rs} \ne 0.0$, $\texttt {tc\_tl}=0.1$, $\texttt {tc\_a5}=0.1$, $\texttt {tc\_a3}=0.1$, $\texttt {tc\_es}=0.2$, $\texttt {tc\_me}=0.1$, and $\texttt {tc\_ir}=0.1$) and constants factors values ($\texttt {k\_tc}$ and $\texttt {c\_s\_r}$) are given such that $\texttt {k\_tc} \times \texttt {c\_s\_r} = 1$ where $\texttt {c\_s\_r}$ is the length of the branch. For instance, regarding transcripts in Gene2, the number of transcripts undergoing the intron retention event is equal to 1, which corresponds to the ceiling result of $\texttt {k\_tc} \times \texttt {c\_s\_r} \times n \times \texttt {tc\_ir}$, where $n=7$ represents the number of source transcripts at this particular evolutionary stage ({**1#1**, **2#0**, **2#2**, **2#3**, **2#4**, **2#5**, **2#6**})

Alternative splicing (AS) is an important mechanism that contributes to the expansion of transcript diversity in eukaryotes [1, 2]. It allows distinct transcripts to be produced from the same gene and plays an important role in the regulation of gene expression. Several studies have shown that numerous human transcripts are conserved across various species [3–6]. For illustration, Fig. 1 shows a simulation example in which the transcript **1#1** of the ancestral gene Gene1 is conserved in descendant genes Gene2 and Gene3 as transcripts **2#1** and **3#1** respectively, composed of exon1, exon2 and exon3.

Despite considerable investigation into the conservation of transcripts and proteins among genes across various species, there is still limited knowledge regarding their evolution. Studying the evolution of transcripts is essential for their annotation, enabling the quantification of isoform-level expression and providing insights into their divergence or conservation across genes and species [7–10]. Moreover, understanding transcript evolution is essential for understanding alternative splicing mechanisms and its impact at the protein structure level. Transcript evolutionary histories help in assessing the functions of transcripts and their homologs, thereby uncovering the evolution of protein function [11]. It also aids in predicting the functions of uncharacterized genomic elements and identifying potential disease markers, thereby shedding light on the molecular basis of pathologies such as cancers and genetic disorders.

However, estimating the evolutionary history of transcripts remains difficult [7–9], and evaluating computational methods developed for this task requires gold standard data. The latter is also needed for the experimental assessment of methods developed for the analysis of spliced RNA sequences, such as splice site prediction [12, 13], identification of homologous exons [5, 14], spliced alignment [15, 16], multiple sequence alignment [17, 18], or splicing orthology inference [19]. In the absence of real gold standard data, there is a need for simulation tools to generate synthetic data. For this purpose, SimSpliceEvol1 [20] was developed in 2019 to generate datasets of simulated gene evolution accounting for alternative splicing.

SimSpliceEvol1 starts by simulating an ancestral gene with its exon-intron structure and a set of alternative transcripts at the root of a guide gene tree. These alternative transcripts differ from each other by their exon compositions. The nucleotide sequences of exons and introns data from the Ensembl coding gene dataset [2] were used to build Markov chains to simulate exon and intron sequences. Subsequently, two evolutionary models are concurrently employed to simulate the evolution of genes along the branches of the guide tree. The first model is for the evolution of the exon-intron structure of the genes and the resulting sets of alternative transcript sequences successively. The second model is a codon/nucleotide sequence evolution model, considering substitution and indel events for both exon sequences at the codon level and intron sequences at the nucleotide level. The evolution model of exon-intron structures comprises three elementary events, exon loss, gain, and duplication, as described in [7]. It is worth noting that SimSpliceEvol1 does not introduce a novel sequence evolution simulation model but instead uses the same models as indel-Seq-Gen for coding exon and non-coding intron sequences [21]

However, SimSpliceEvol1 does not integrate a model of sets of transcripts evolution. Thus, the sets of transcripts simulated at distinct nodes of the guide gene tree are not related by an evolutionary history. This limitation makes SimSpliceEvol1 useless for testing methods for transcript phylogeny inference, as well as methods for gene phylogeny inference that rely on transcript conservation. We have developed SimSpliceEvol2 to address this need. It extends SimSpliceEvol1 by explicitly incorporating a model of transcript evolution, thus enabling the simulation of the evolution of sets of transcripts along the branches of a guide gene tree. Moreover, we provide a new standalone graphical user interface in addition to the updated web server for easy and user-friendly access to the tool.

## Implementation

### Inputs description

SimSpliceEvol2 takes the same inputs as SimSpliceEvol1. Namely, it requires a guide gene tree with branch lengths in the NHX or Newick format. Here, a guide tree refers to the topology of a gene tree, representing the evolution of a gene family with gene labels at the leaves of the tree and including branch lengths. The branch lengths are used to calculate the number of mutation events acting on the gene sequences, the exon-intron structures of genes, and on the sets of transcripts produced from genes during the evolution on each branch of the gene tree [22, 23].

There are four categories of optional user-defined parameters. For space reasons, in this sequel, we recall only the notations for the optional parameters that are used in the simulation of transcript evolution. A comprehensive explanation of each user-defined parameter and their impact on the simulated data is available at https://simspliceevol.cobius.usherbrooke.ca. The first category of optional parameters includes constant factors used to compute the number of mutation events on branches of the gene phylogeny. In particular, the user-defined constant $\texttt {k\_tc}$ helps to determine the number of events that change the set of transcripts produced from a gene.

The second and third categories of optional parameters include those that are used in the simulation of exon sequence evolution and the simulation of exon-intron structure evolution.

We now describe in detail the fourth category, which includes parameters used to compute the number of each type of event acting on the set of transcripts produced from a gene. There are seven such types of events including five types of alternative splicing events, plus a transcript loss event, and a transcript gain event. The five types of alternative splicing events, which explain the differences between any two transcripts of a gene are alternative 3’ (a3) or 5’ (a5) splice-site selections; Exon skipping (es); Mutually exclusive (me) exons; and Intron retention (ir). Thus, the seven parameters in this category are denoted by $\texttt {tc\_a5}$, $\texttt {tc\_a3}$, $\texttt {tc\_es}$, $\texttt {tc\_me}$, $\texttt {tc\_ir}$, $\texttt {tc\_tl}$, and $\texttt {tc\_rs}$, corresponding respectively to the seven type of mutation events that can change the set of transcripts produced from a gene. They define the relative proportion, respectively, of alternative splicing events, namely of types a5, a3, es, me, and ir, of transcripts randomly selected (rs) or in others terms gained among all possible isoforms, and of transcripts lost (tl). For instance, the anticipated total number of transcripts undergoing es events for a gene with *n* transcripts on a branch of the gene tree with a length of $\texttt {c\_s\_r}$ is given by the formula $n \times \texttt {tc\_es} \times \texttt {k\_tc} \times \texttt {c\_s\_r}$.

Like SimSpliceEvol1, SimSpliceEvol2 maintains a large number of user parameters to enable users to simulate and test various frequencies for the evolutionary events.

### Transcript evolution framework

SimSpliceEvol2 introduces improvements in simulating the evolution of sets of transcripts compared to SimSpliceEvol1.

#### Simulation of the set of transcripts at the root of the guide tree

Considering the guide tree, the simulation of the set of transcripts at the root of the tree is operated in two steps. In the first step, transcripts are generated by randomly selecting them from all potential transcripts isoforms (all $2^m -1$ possible combinations of exons, where *m* is the number of exons in the gene structure) at the root. If $tc_{rs}$ is *null*, then only one transcript, composed of all exons in the gene structure, is selected. Otherwise, a heuristic is used for the random selection of transcripts in the pool of $2^m$ possible isoforms. This heuristic first involves the random selection of a number *n* of transcripts, following a normal distribution with a mean of 1.45 and a standard deviation of 1.08. These parameter values were determined in [20] and correspond to the mean number of transcripts per gene and the standard deviation of transcripts per gene in the Eukaryote kingdom based on data from the Ensembl Compara database [24]. In the second step, the remaining transcripts are produced by applying AS events to the transcripts randomly selected in the first step. Note that, a transcript can undergo more than one AS event during the simulation. Figure 1 provides an illustration. At the root of the tree, the randomly selected transcripts are **1#1** and **1#2**, and both underwent a sequence of AS events, for example, giving rise to transcripts **1#4**, **1#5** and **1#7** from **1#1**, and to **1#3** and **1#6** from **1#2**. For example, the transcript **1#6** is derived from transcript **1#2** by applying first an *es* event to yield **1#3**, and then an a3 event to yield **1#6**. The loss of transcripts does not occur at the root of the tree, assuming the root represents the starting point of evolution of a set of homologous transcripts. Consequently, all relative proportions of AS are directly applied to the overall count of existing transcripts at a given evolutionary stage. An evolutionary stage is defined as the period between a time where a set of source transcripts are available for a gene, and the time where a new set of sink transcripts are available after applying mutation events to the source transcripts, as illustrated in Fig. 1. Each sink transcript is then linked to at most one source transcript from which it is derived. For example in Fig. 1, at the root of the gene tree, the number of transcripts undergoing the *es* event is determined by taking the ceiling value of $n \times tc\_es$, where $n=2$ represents the number of source transcripts at this particular stage of evolution, and $tc\_es=0.3$. In this case, only one transcript is subject to *es* event and the next stage of evolution then has a total of 3 source transcripts. This approach is used to determine the quantity of transcripts undergoing a given type of AS event at each evolutionary stage throughout the simulation.

#### Simulation of the set of transcripts at other nodes than the root

Considering an internal node or a leaf node of the guide gene tree, the exon-inton structure of this node is compared to the exon-intron structure of its parent node. First, a transcript loss is inferred for all transcripts of the parent node for which one or multiple exons were lost on the branch between the parent gene node and the child gene node. The remaining transcripts are denoted as conserved transcripts. For example, in Fig. 1, the transcripts **1#1** and **1#7** from gene Gene1 are conserved on the branch leading to Gene2, while transcripts **1#2**, **1#3**, **1#4**, **1#5**, and **1#6** are lost because of the absence of exon4 in the structure of Gene2.

Subsequently, the conserved transcripts continue their evolution. A conserved transcript may be lost, since another way to loose transcripts is regulated by the user-defined parameter $\texttt {tc\_tl}$. For instance, in Fig. 1, the transcript **1#7** is initially conserved at the start of the branch between Gene1 and Gene2, but it is finally lost in the next evolutionary stage. After these steps, transcripts randomly selected from all potential transcript isoforms are added to the set of conserved transcripts. The remaining transcripts are then generated by applying AS events to the set of available transcripts, following a process similar to the one used at the root of the guide tree. However, the process now takes into account the length $\texttt {c\_s\_r}$ of the branch between the parent and child node in the guide gene tree, and it also accounts for the constant factor $\texttt {k\_tc}$ provided by the user. For instance, in Fig. 1, the number of transcripts undergoing *es* on the branch from Gene1 to Gene2 equals the ceil of $\texttt {k\_tc} \times \texttt {c\_s\_r} \times n \times \texttt {tc\_es}$, where *n* represents the number of source transcripts at the given evolutionary stage. The result is 1 given $\texttt {k\_tc} \times \texttt {c\_s\_r} = 1$, $\texttt {tc\_es}=0.2$ and $n=3$.

The simulation framework adheres to the Dollo parsimony principle, which is that an exon cannot be gained during the evolution after being lost. At the end of the simulation process, SimSpliceEvol2 yields a forest of transcript trees that describe the evolutionary history of transcripts in the gene family. In contrast to SimSpliceEvol1, which mainly focussed on generating sequences of alternative transcripts at the leaves of the guide tree, SimSpliceEvol2 explicitly infers the transcript phylogeny.

### Software design

#### Web server

**Fig. 2:**
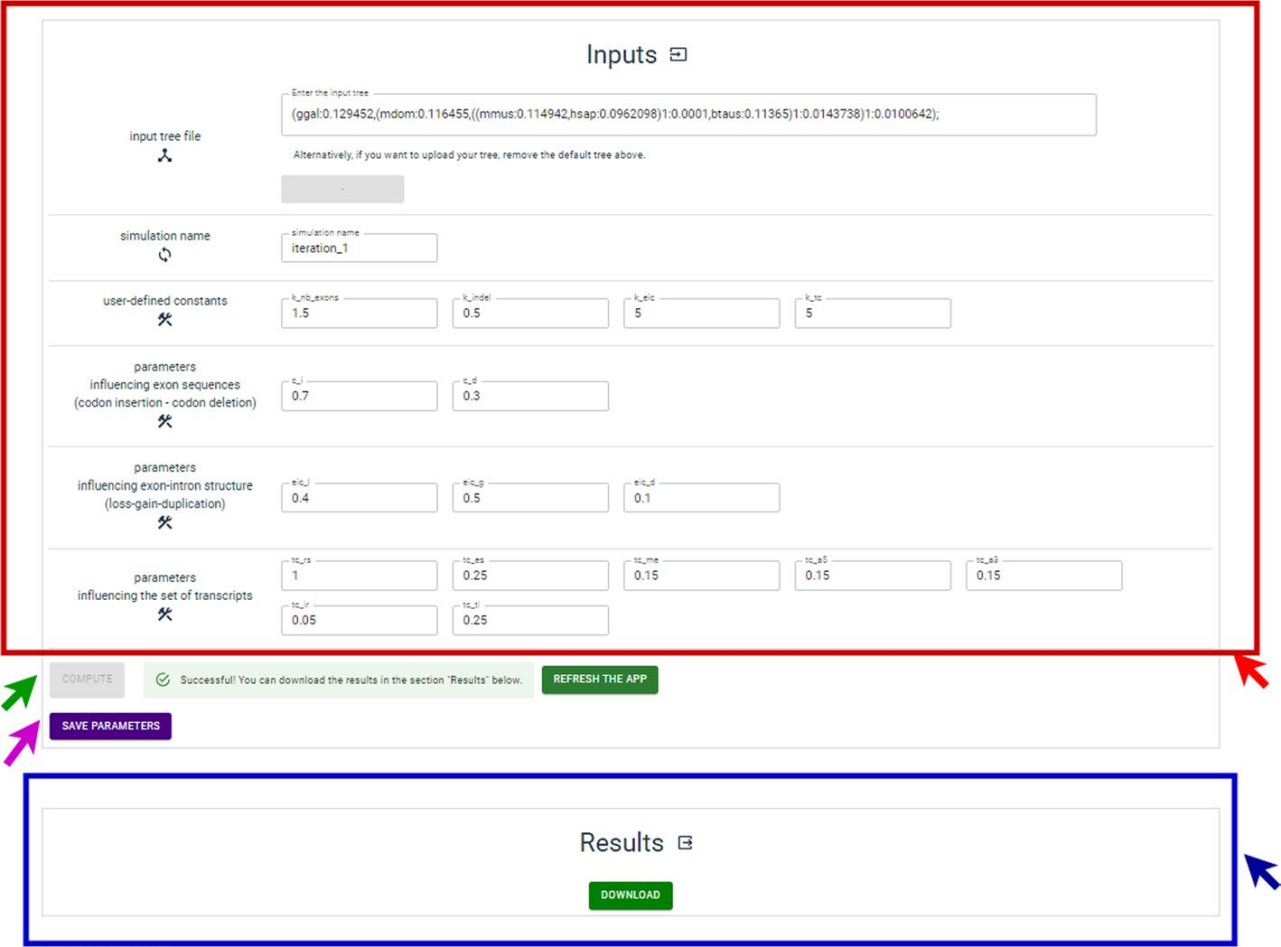
Web server screenshot. The screenshot presents two main sections of the web server. The input section, highlighted in red, allows users to set parameter values. Users can launch the program (illustrated by a green arrow) and save the query parameters for future use (illustrated by a purple arrow). The results section, highlighted in blue, displays the available data for download once it is ready, as indicated by the blue arrow

The current web server was implemented by deploying a Linux-based Apache 2.4.41 Web server on a Ubuntu 20.04.6 LTS system. It is fully compatible with standard desktop PC systems. We have updated the user interface of the web server provided for SimSpliceEvol2, to make it more user-friendly. Now, the Web interface is constructed using the ViteJS 4.1 framework. Users can now manually enter their input guide tree, in addition to the option to upload it as a file. Additionally, users now have the possibility to save their query parameters for future reference and analysis. Once users have completed the parameters, they can launch the simulation by using the “compute” button. When the simulation is finished, users can download the simulated datasets available in the “results” section, as illustrated in the Fig. 2. The web server is available at https://simspliceevol.cobius.usherbrooke.ca.

#### Standalone software and graphical user interface

**Fig. 3:**
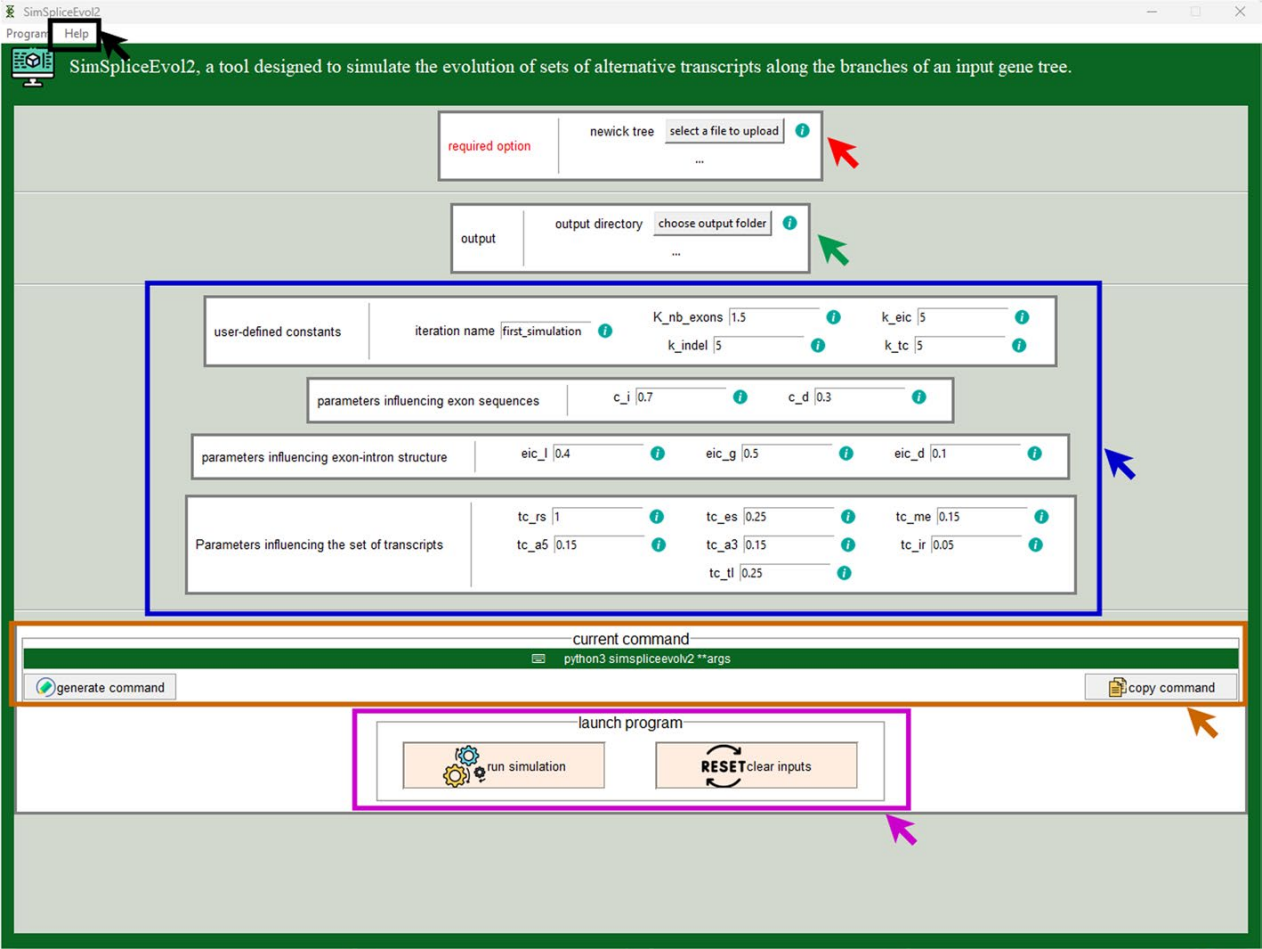
SimSpliceEvol2 GUI screenshot. The graphical user interface of SimSpliceEvol2 enables users to browse the file system for selecting the input guide tree file (indicated by the red arrow) and the output directory (indicated by the green arrow). It enables also to set parameter values (indicated by the blue arrow). Upon clicking the “generate command” button, the corresponding command line is produced (indicated by the orange arrow). Users have the option to copy this command for future use by clicking the “copy command” button. Help related to the program or its options is provided at the top of the interface (indicated by the black arrow). The outputs generated from running the simulation (indicated by the purple arrow) correspond to the figures shown in Fig. 4

**Fig. 4:**
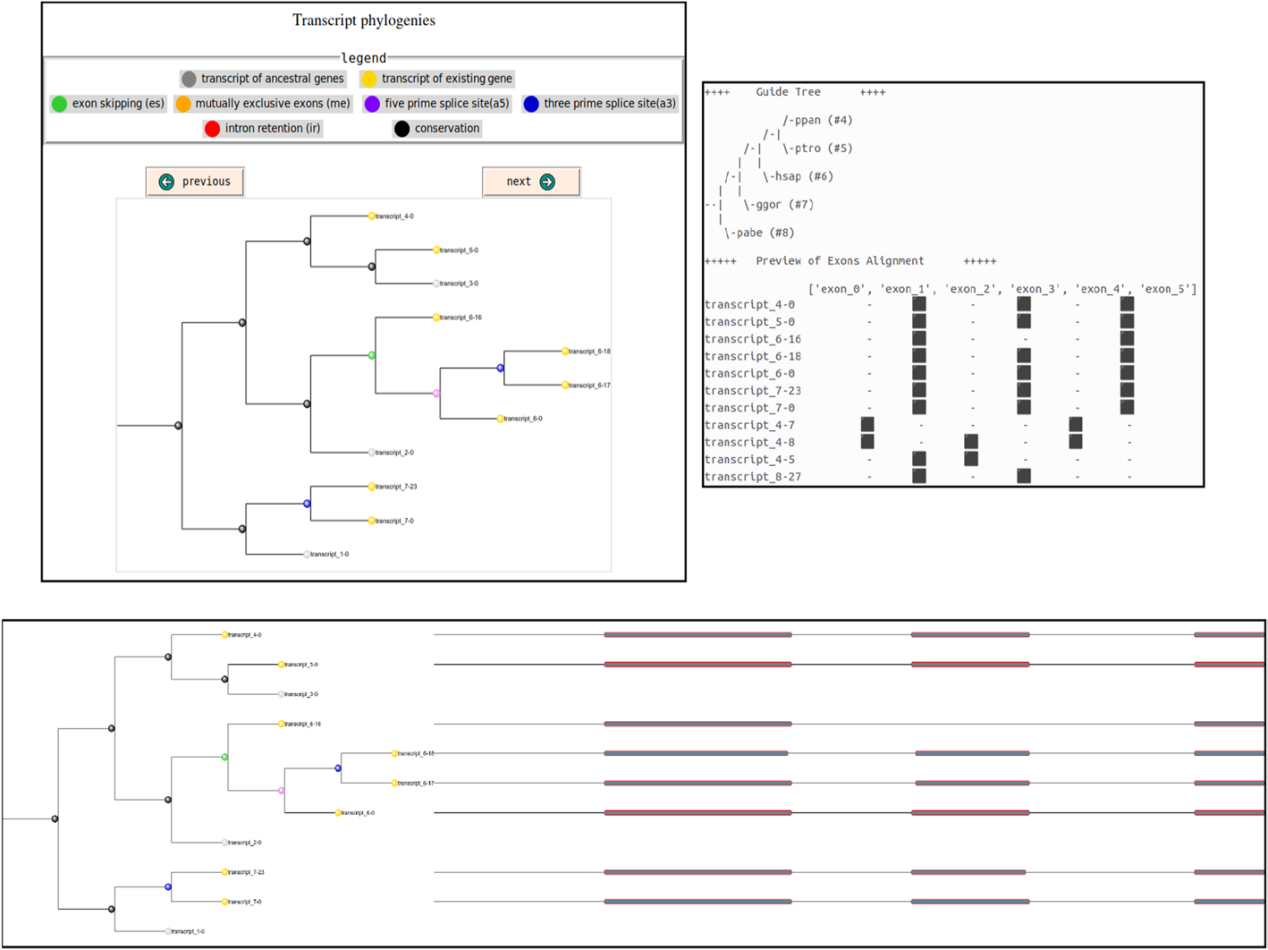
Outputs of SimSpliceEvol2 using the GUI with the default options (k_tc=5, tc_rs=1, tc_es=0.25, tc_me=0.15, tc_a5=0.15, tc_a3=0.15, tc_ir=0.15, tc_tl=0.05). (**Top left**) Visualization of the simulated transcript phylogeny (multiple transcript trees that form a transcript forest) through a carousel interface within the GUI (figures generated using ETE 3 [25]). (**Top right**) An exon alignment for all transcripts generated at leaves of the phylogeny is shown alongside the guide gene tree. (**Bottom**) The software outputs figures that show the multiple sequence alignment of transcripts generated at the leaves of each transcript tree (figure also generated using ETE 3 [25])

We have improved the software by integrating a model of transcript evolution, as detailed in Sect. 2.2. We have included it within a standalone Graphical User Interface (GUI) in order to distribute a single software, as shown in Fig. 3. The software is available for download at https://simspliceevol.cobius.usherbrooke.ca. It is a standalone application designed to run the simulation program locally without any dependencies. Currently, it has been developed and tested on Linux (Ubuntu 22.04.3 LTS and later versions) and Windows 11 operating systems, and no user login is required. SimSpliceEvol2 allows to generate figures that display the transcript phylogenies, together with the corresponding multiple sequence alignment. For example, in Fig. 4 bottom, the phylogenetic tree is diplayed with leaf colors that highlight, in yellow, a group of orthologous transcripts, including transcript_4-0, transcript_5-0, transcript_6-0, transcript_7-0, alongside the multiple sequence alignment of the transcripts present in the tree. Referring to Fig. 1, two transcripts are orthologs if they are derived from distinct genes and there are no AS events in the path of branches connecting them, i.e., the path contains only black edges in the figure. A group of orthologous transcripts is a set of transcripts which are pairwise orthologs. In Fig. 1, the only group of orthologous transcripts is $\{{\textbf {2\#1}}, {\textbf {3\#1}}\}$. For more detailed documentation on the software, the reader is invited to consult https://simspliceevol.cobius.usherbrooke.ca.

SimSpliceEvol2 improves the output of SimSpliceEvol1 by incorporating a visualization component through its GUI, and by providing more relevant data for transcript phylogeny analysis. Within the GUI, a carousel window is provided to assist users in exploring phylogenies. Additionally, the software displays the alignment of transcripts to illustrate the exon structure conservation between all simulated transcripts.

## Results

**Fig. 5:**
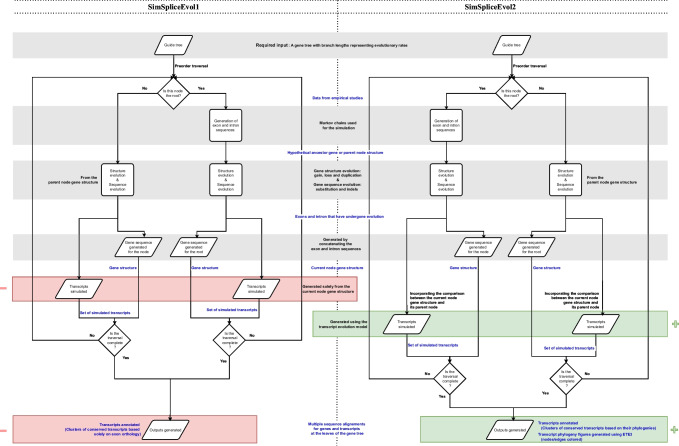
Workflows of SimSpliceEvol1 and SimSpliceEvol2. The illustration compares SimSpliceEvol1 and SimSpliceEvol2 to emphasize the new transcript evolution model integrated. Changes from SimSpliceEvol1 are indicated in red (with a red minus sign), while additions from SimSpliceEvol2 are depicted in green (with a green plus sign). Tasks retained by both methods are underscored in gray and the data flow is highlighted in blue

In Fig. 5, we illustrate the workflows of both tools, SimSpliceEvol1 and SimSpliceEvol2, to highlight the different processes employed and to contextualize the transcript evolution model proposed in SimSpliceEvol2. SimSpliceEvol2 generates an output that comprises the gene sequences located at the leaves of the guide gene tree. The output also includes the transcript sequences associated with each gene at each node of the guide gene tree, by providing details about their exon content. Additionally, the information regarding the start and end locations of exons in both the gene and transcript sequences is provided. SimSpliceEvol2 also outputs all groups of orthologous transcripts [6]. Moreover, SimSpliceEvol2 outputs the phylogeny for all the transcripts at the leaves of the guide tree. This phylogeny consists of a forest of transcript trees, describing the evolutionary history of transcripts. The program also outputs a record of all simulated evolutionary events that occurred along the branches of the guide tree. This information is used to generate and output the multiple sequence alignment of all gene and transcript sequences simulated.

**Fig. 6:**
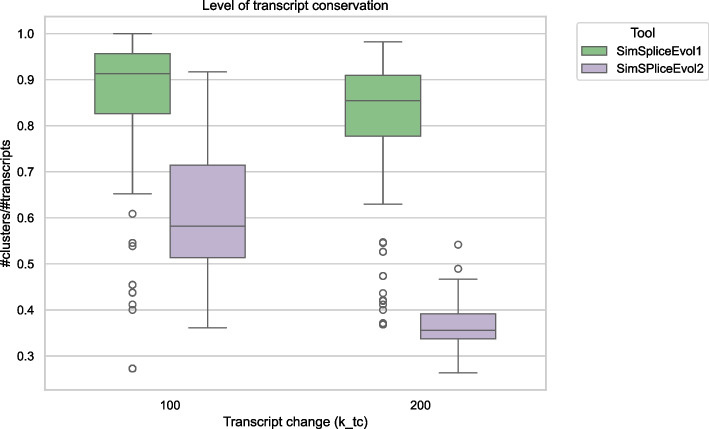
Transcript level conservation in SimSpliceEvol1 and SimpliceEvol2

SimSpliceEvol1 and SimSpliceEvol2 are simulation tools, and since some parts of their simulations rely on random processes, we cannot generate a common simulated dataset to directly compare them. However, we can compare the level of transcript conservation between datasets generated by SimSpliceEvol1 and SimSpliceEvol2 for the same input data. Transcript clusters are sets of conserved transcripts. SimSpliceEvol1 identifies these clusters by comparing the composition of exons in transcripts, while SimSpliceEvol2 identifies them based on the transcript phylogenies. Using the same input parameters (tc_rs=1, tc_es=0.25, tc_me=0.15, tc_a5=0.15, tc_a3=0.15, tc_ir=0.15, tc_tl=0.05 with the default tree), we performed two sets of 100 iterations for two values of the parameter k_tc (k_tc = 100 and k_tc = 200) with SimSpliceEvol1 and SimSpliceEvol2. We then calculated the ratio of the number of conserved clusters to the number of transcripts generated in each iteration. A ratio of 1 indicates low conservation of transcripts, as no transcripts are grouped together in a cluster, while a ratio that tends toward 0 indicates that the transcripts are all grouped into a single cluster. A higher value of k_tc will result in more alternative splicing (AS) events, leading to the generation of numerous transcripts. Since SimSpliceEvol2 makes the transcripts evolve through a process that integrates transcript conservation, we expect the ratio to be lower for SimSpliceEvol2 than for SimSpliceEvol1 in the two datasets. In Fig. 6, we can see that the ratios are mostly higher than 0.6, with an approximate average around 0.9 for SimSpliceEvol1. In contrast, for SimSpliceEvol2, the ratios are significantly lower. Therefore, based on the same inputs, SimSpliceEvol1 clearly generates less transcript conservation than SimSpliceEvol2. Additionally, SimSpliceEvol2 still presents a higher level of transcript conservation than SimSpliceEvol1, even when the number of transcripts is increased due to the increase in the parameter k_tc value.

## Discussion

For the second version of SimSpliceEvol, we focus on the development of the model for transcript evolution. To the best of our knowledge, SimSpliceEvol2 is the only tool available for simulating synthetic transcript phylogenies.

Since we generate synthetic transcript phylogenies and transcript sequence alignments using SimSpliceEvol2, these data can be considered as ground truth to evaluate the accuracy of methods to reconstruct transcript phylogenies or to compute transcript sequence alignments. In this context, the synthetic data are called “true” and can be compared with the data estimated by other methods.

SimSpliceEvol2 was used in [10] to assess the robustness and precision of a new method for transcript phylogenies reconstruction. In [10], the robustness of the method to alignment methods was assessed by comparing phylogenies computed using estimated alignments with those derived from the true sequence alignments provided by SimSpliceEvol2. Subsequently, the precision of the method was evaluated by comparing the resulting phylogenies with the true phylogenies generated by SimSpliceEvol2. We also used the software to assess the precision of a method described in [6] for identifying orthologous transcripts. The software was also utilized in [18] to generate synthetic transcript datasets. These datasets were employed to assess the performance of a novel spliced alignment method.

We are aware that the synthetic data generated by SimSpliceEvol2 could be improved, as it lacks functional meaning because of the independent generation of codon sequences. Additionally, it is important to note that the application of an a5 or an a3 event always induces a nucleotide segment whose length is a multiple of 3. Furthermore, the length of a transcript sequence is also a multiple of 3 because we only consider the Coding DNA sequence.

In some cases, a simulated transcript may lack an initiation codon and/or stop codon. Such transcripts would not be classified as protein-coding transcript, as we define protein-coding transcript to begin with an initiation codon and end with a stop codon. This result is achieved randomly during the simulation process.

Therefore, it becomes imperative to introduce specific splice site evolution model to regulate alternative splicing events for transcripts, particularly for a3 and a5 events. Although our focus is solely on coding DNA sequences and we do not propose a model of intron evolution, in future works, it will be important to integrate a model for intron evolution comprising intron gain, loss, and sliding events, as mentioned in [3, 26, 27].

## Conclusion

SimSpliceEvol2 improves on SimSpliceEvol1 by incorporating a new important feature that regards the model of transcripts evolution. An updated version of the SimSpliceEvol web server and a new user-friendly GUI are provided to facilitate the use of the simulation tool. Additionally, the output of SimSpliceEvol was improved by adding visualization components. In particular, the GUI provides a carousel that allows to visualize all the transcript phylogenies generated by the software, thus enhancing the analytical experience of the user. In summary, SimSpliceEvol2 generates synthetic datasets that are valuable for the purposes of benchmarking and method assessments. The web server is accessible at https://simspliceevol.cobius.usherbrooke.ca, where you can also download the standalone software. Comprehensive documentation for the software is available at the same address.

## Availability and requirements

- **Project name:** SimSpliceEvol2
- **Project home page:** https://simspliceevol.cobius.usherbrooke.ca
- **Web server & Software-GUI:** https://simspliceevol.cobius.usherbrooke.ca
- **Operating systems:** Linux (Ubuntu 22.04.3 LTS and later versions) & Windows 11
- **Programming language:** Python
- **Other requirements: ** Python 3.6 or higher; ETE3 (version 3.1 at least); pyQt5 (version 5.8 at least); pandas (version 2.2 at least); numpy(version 1.26 at least)
- **License:** GPL
- **Any restrictions to use by non-academics:** License needed

## Data Availability

Web server & Software-GUI accessible at: https://simspliceevol.cobius.usherbrooke.ca. Source code available at: https://github.com/UdeS-CoBIUS/SimSpliceEvol.

## Abbreviations

AS: Alternative Splicing
a3: Alternative 3’ splice site
a5: Alternative 5’ splice site
DNA: DeoxyriboNucleic Acid
es: Exon Skipping
GUI: Graphical User Interface
ir: Intron Retention
me: Mutually Exclusive exons
NHX: New Hampshire eXtented format
rs: Random Selection
tc: Transcript Change
tl: Transcript Loss

## References

[1] Harrow J (2012). GENCODE: the reference human genome annotation for The ENCODE Project. Genome Res.

[2] Zerbino DR, Achuthan P, Akanni W, Amode MR, Barrell D, Bhai J (2018). Ensembl 2018. Nucleic Acids Res.

[3] Keren H, Lev-Maor G, Ast G (2010). Alternative splicing and evolution: diversification, exon definition and function. Nat Rev Genet.

[4] Guillaudeux N, Belleannée C, Blanquart S (2022). Identifying genes with conserved splicing structure and orthologous isoforms in human, mouse and dog. BMC Genomics.

[5] Ma J, Wu JY, Zhu L (2022). Detection of orthologous exons and isoforms using EGIO. Bioinformatics.

[6] Ouedraogo WYDD, Ouangraoua A. Inferring Clusters of Orthologous and Paralogous Transcripts. In: RECOMB-CG. Springer; 2023. p. 19–34.

[7] Christinat Y, Moret BM. Inferring transcript phylogenies. In: 2011 IEEE International Conference on Bioinformatics and Biomedicine. IEEE; 2011. p. 208–215.

[8] Christinat Y, Moret BME (2013). A transcript perspective on evolution. IEEE/ACM Trans Comput Biol Bioinf.

[9] Ait-Hamlat A, Zea DJ, Labeeuw A, Polit L, Richard H, Laine E (2020). Transcripts’ evolutionary history and structural dynamics give mechanistic insights into the functional diversity of the jnk family. J Mol Biol.

[10] Ouedraogo WYD, Ouangraoua A. Inferring Transcript Phylogenies from Transcript Ortholog Clusters. In: RECOMB International Workshop on Comparative Genomics. Springer; 2024. p. 47–68.

[11] Black DL (2000). Protein diversity from alternative splicing: a challenge for bioinformatics and post-genome biology. Cell.

[12] Goldstein LD, Cao Y, Pau G, Lawrence M, Wu TD, Seshagiri S (2016). Prediction and quantification of splice events from RNA-seq data. PLoS ONE.

[13] Scalzitti N, Kress A, Orhand R, Weber T, Moulinier L, Jeannin-Girardon A (2021). Spliceator: multi-species splice site prediction using convolutional neural networks. BMC Bioinform.

[14] Siepel A, Haussler D. Computational identification of evolutionarily conserved exons. In: Proceedings of the Eighth Annual International Conference RECOMB. RECOMB ’04. New York, NY, USA: ACM; 2004. p. 177–186.

[15] Jammali S, Aguilar JD, Kuitche E, Ouangraoua A (2019). SplicedFamAlign: CDS-to-gene spliced alignment and identification of transcript orthology groups. BMC Bioinform.

[16] Reinhardt F, Stadler PF (2022). ExceS-A: an exon-centric split aligner. J Integr Bioinform.

[17] Kapustin Y, Souvorov A, Tatusova T, Lipman D (2008). Splign: algorithms for computing spliced alignments with identification of paralogs. Biol Direct.

[18] Jammali S, Djossou A, Ouédraogo WYD, Nevers Y, Chegrane I, Ouangraoua A (2022). From pairwise to multiple spliced alignment. Bioinform Adv..

[19] Blanquart S, Varré JS, Guertin P, Perrin A, Bergeron A, Swenson KM (2016). Assisted transcriptome reconstruction and splicing orthology. BMC Genomics.

[20] Kuitche E, Jammali S, Ouangraoua A (2019). SimSpliceEvol: alternative splicing-aware simulation of biological sequence evolution. BMC Bioinform.

[21] Strope CL, Abel K, Scott SD, Moriyama EN (2009). Biological sequence simulation for testing complex evolutionary hypotheses: indel-Seq-Gen version 2.0. Mol Biol Evol..

[22] Barbosa-Morais NL, Irimia M, Pan Q, Xiong HY, Gueroussov S, Lee LJ (2012). The evolutionary landscape of alternative splicing in vertebrate species. Science.

[23] Kim E, Magen A, Ast G (2007). Different levels of alternative splicing among eukaryotes. Nucleic Acids Res.

[24] Martin FJ, Amode MR, Aneja A, Austine-Orimoloye O, Azov A, Barnes I (2022). Ensembl 2023. Nucleic Acids Res..

[25] Huerta-Cepas J, Serra F, Bork P (2016). ETE 3: reconstruction, analysis, and visualization of phylogenomic data. Mol Biol Evol.

[26] Rogozin IB, Carmel L, Csuros M, Koonin EV (2012). Origin and evolution of spliceosomal introns. Biol Direct.

[27] Stoltzfus A, Logsdon JM, Palmer JD, Doolittle WF (1997). Intron “sliding” and the diversity of intron positions. Proc Natl Acad Sci.

